# Spatial distribution evolution and accessibility of A-level scenic spots in Guangdong Province from the perspective of quantitative geography

**DOI:** 10.1371/journal.pone.0257400

**Published:** 2021-11-15

**Authors:** Zhenjie Liao, Lijuan Zhang

**Affiliations:** School of Management, Guangzhou Huashang College, Guangzhou, China; Northeastern University, CHINA

## Abstract

As a typical representative of tourism resources, the spatial distribution of A-level scenic spots has a profound impact on the layout of tourism industry. Scenic spot accessibility is also important for the development of tourism. However, the relationship of regional accessibility and spatial distribution of A-level scenic spots are understudied. The study used quantitative geography and geographic information system spatial analysis methods and analyzed the evolution of spatial distribution and regional accessibility of A-level scenic spots in Guangdong Province from 2001 to 2020. The results present the following: 1. Agglomeration distribution is the main distribution type of A-level scenic spots in Guangdong Province, and the spatial distribution is unbalanced. 2. From 2001 to 2020, the spatial distribution of A-level scenic spots in 21 prefecture-level cities of Guangdong Province has gradually developed from "wide gap" to "relatively reasonable." 3. Distribution density of A-level scenic spots in Guangdong Province has evolved into the main core area of high density. 4. Center of the gravity of A-level scenic spots in Guangdong Province developed from east to west during 2002–2007 and moved to the east after 2007. 5. Accessibility between A-level scenic spots and tourist source areas in Guangdong Province is good, with an evident aggregation phenomenon. This study reveals the spatial distribution evolution law and regional accessibility of A-level scenic spots, which is conducive to healthy, sustainable, and stable development of tourism in Guangdong Province.

## Introduction

Tourism administration is a complex system, in which the construction of scenic spots is the prerequisite for the development of tourism, the space carrier of tourism activities, and the core part of tourism products [1]. As a core component of tourism, scenic spots and their spatial distribution and accessibility not only determine the spatial behavior of tourists, but also profoundly affect the formulation of regional tourism development strategies [2]. Therefore, research on scenic spots has been highly valued regionally and globally [3]. In addition, an expansion in the research of methodologies has occurred, especially related to the interpretation and classification [4,5], competition [6,7], tourists’ perception [8,9], and spatial distribution and evolution [10–12] of scenic spots.

In China, A-level scenic spots include grade A, 2A, 3A, 4A and 5A, of which 5A grade is the highest level. The China National Tourism Administration (CNTA) started to implement the system of classifying and rating the quality of tourist scenic spots in 1999, and A-level scenic spots officially appeared in 2001, and the number of A-level tourist scenic spots has been increasing ever since. A-level scenic spots are considered an important part of China’s tourism industry [13], and are recognized as one of the most important travel destinations for tourists. The regional spatial structure of A-level scenic spots is not only an indicator of the industry’s response to tourism demand but is also deemed an indicator of performance of the continuous improvement of tourism supply levels [14,15]. The distribution of A-level scenic spots in a region directly reflects the tourism industry’s development in the region [16,17]. A-level scenic spots representing quality and standard play an important role in cultivating tourism products, improving the competitiveness of scenic spots, and promoting local economic development, and a correct understanding of the differences in spatial distribution and accessibility of A-level scenic spots is a fundamental task to determine the optimal allocation of tourism resources and development [18]. Spatial distribution is an interactive relationship produced by the joint action of tourism elements and spatial geography [19], and is the result of a gradual accumulation of tourism in the process of long-term development and can provide a reference for spatial query of the research area [20]. The related research content is mostly about the internal relationship of space [21], the relationship between spaces [22,23], and the relationship between space and external development [24,25]. Research on the spatial structure of A-level scenic spots is currently focused on two aspects. First, the spatial structure and distribution law [26]. The spatial distribution characteristics [27], patterns, and influencing factors of A-level scenic spots are analyzed [28,29]. This includes spatial structure, differences, and distribution characteristics of A-level scenic spots analyzed from national, regional, provincial, and municipal perspectives. Research on the spatial distribution of A-level scenic spots is primarily based on relevant quantitative geography methods [30]. Other methods include geographic information system (GIS) spatial analysis and the economic geography model [31,32]. Most studies are based on the point axis theory, the core-edge theory, and the tourism destination life cycle theory. The methods primarily adopted include GIS analyses and mathematical models, and the majority are based on quantitative analysis. In general, considerable research on the spatial distribution of scenic spots has been conducted regionally and globally. Second, the spatial distribution evolution of A-level scenic spots to reveal the dynamic change process of scenic spot distribution [33]. The main purpose is to analyze the evolutionary characteristics of the spatial structure of A-level scenic spots in the study area in multiple temporal cross-sections, using resources such as A-level scenic spots in certain regions as the research object [34]. From a comprehensive perspective, most scholars use scenic area data of a single year to explore the spatial differentiation pattern and characteristics of a certain level from a static perspective. In contrast, the change process from the dynamic perspective of time series and what evolutionary patterns and mechanisms of action are presented in the spatial distribution of different regions and different levels of scenic areas are rarely addressed [35]. Hitherto, research into the spatial distribution of A-level scenic spots remains limited to metropolitan areas and certain provinces [36]. Moreover, no study has been conducted on the spatial distribution evolution of A-level scenic spots in Guangdong Province from 2001 to 2020.

On the other hand, accessibility is an important factor to ensure the sustainability of A-level scenic areas. Accessibility refers to the number of research objects that are accessible and approachable at a spatial scale [37], and is usually used as a research criterion in terms of time or spatial distance. Accessibility as a valid indicator of the performance of transportation network structures was first proposed by Hansen [38] and is not uniformly settled in academia owing to its broad scope. The best accepted accessibility is based on a specific expression of the ability of individuals or groups of transportation systems to move within a region. At present, scholars are characterized by multiple fields and methods in the study of accessibility, and the research involves traffic geography, urban geography, land use, economic geography, and other fields [39–41], mainly applying the distance metric, contour, topological network connection, and potential model methods [42,43]. However, there are few studies on the accessibility of A-level scenic spots. These studies mainly involve the measurement of accessibility of A-level scenic spots at different spatial scales [44–46] and the impact of certain transportation mode changes (high-speed rail, etc.) on the accessibility of A-level scenic spots [47]. In addition, the applied methods are also mainly limited to buffer zone analysis [48], transportation cost weighted values [49], and raster cost weighted distance algorithms [50].

Because accessibility can well reflect the topographic and geomorphological characteristics of a region and the ease of reaching it, in this work, we used the GIS spatial analysis method to quantitatively analyze the current spatial distribution and regional accessibility of A-level scenic spots to explore the relationship between the spatial distribution evolution of A-level scenic spots in Guangdong Province and the natural environment in which they are located. Our results provide a basis for further development of tourism in Guangdong Province and allow the sustainable utilization of resources.

## Materials and methods

### Data sources

The list of A-level scenic spots in Guangdong Province was primarily obtained from the Department of Culture and Tourism of Guangdong Province. The information relating to scenic spots is also published on government or Tourism Administration websites for 21 prefecture-level cities in Guangdong Province. The information regarding the time of evaluation for each of the A-level scenic spots in Guangdong Province is provided at the National Tourism Administration government website; the relevant notice announcement is published by the Department of Culture and Tourism of Guangdong Province, and the information at the time of evaluation for a few 3A and 2A scenic spots is sourced from government websites and from the Tourism Administration websites of various cities. To determine the time of evaluation of individual scenic spots, relevant news reports were referred to, or telephonic or on-the-spot investigations to the staff at scenic spots was conducted. The 1:500000 traffic map and the 1:500000 administrative district map of the Guangdong Province Map Compilation Committee of Guangdong Province were used to illustrate the base map containing data on administrative divisions, traffic roads, and rivers considered in this study.

### Data preprocessing

The collected scenic spot data were processed, and a scenic spot database was established. The generated database includes information on the scenic spot names, locations (county and city), grades, their corresponding year of evaluation, and their spatial distributions in Guangdong Province.

### Research methods

#### Average nearest-neighbor index

The average nearest neighbor index (C’) refers to the ratio between the average nearest distance (average observation distance) (C_1_) and the theoretical nearest distance (expected average distance) (C_2_) of each point in the region [51], which is calculated as follows:

$$C_{1}=\frac{\sum_{i=1}^{n}d_{i}}{n},\;C_{2}=\frac{1}{2\sqrt{n/S}},\;C^{'}=\frac{C_{1}}{C_{2}}$$ (1)

where, n represents the number of points which indicates the number of A-level scenic spots in Guangdong Province, s represents the area to be analyzed, and Di represents the Euclidean distance from the scenic spot I (point) to its nearest scenic spot (point). When C’>1, the point elements are uniformly distributed; when C’<1, the point elements are distributed in a cluster.

#### Gini coefficient

The Gini coefficient is typically used to measure the difference in the national economic income in the field of economics. It is a common indicator used for measuring the income gap between the residents in a country or region. Presently, investigations by researchers in China for the calculation and application of various Gini coefficients in the fields of geographical and spatial distribution are underway. In the present study, a simple Gini coefficient calculation method [52] was adopted to accurately analyze the spatial distribution equilibrium of A-level scenic spots in 21 cities of Guangdong Province. The theoretical formula is as follows:

$$Gini=1-\frac{2\sum_{i=1}^{n-1}W_{i}+1}{n}$$ (2)

In Eq (2), Gini represents the Gini coefficient, n represents the number of the scenic spots (21), and Wi represents the proportion of cumulative quantity to total quantity. The Gini value ranges from 0–1. The following can be implied: the higher the Gini value, the more unbalanced and the higher the concentration the spatial distribution of A-level scenic spots is.

#### Nuclear density analysis

Kernel density estimation (KDE) is based on the assumption that geographical events can occur at any location in space; however, the probability of the occurrence is different at different locations. The probability of an event occurrence is high in areas with dense points but is low in areas with sparse points. Nuclear density reflects the distribution quantity, overall distribution orientation, and accumulation distribution area of point elements in a certain area. In the present study, the A-level scenic spots in Guangdong Province were considered as point elements, and more specific distribution changes could be obtained by analyzing the annual nuclear density [53]. The theoretical formula for nuclear density is as follows:

$$f_{n}(x)=\frac{1}{nh}\sum_{i=1}^{n}k(\frac{x-X_{i}}{h})$$ (3)

where, $k(\frac{x-X_{i}}{h})$ represents the kernel function, H represents the bandwidth, and H > 0. (x-Xi) represents the distance from the scenic spot x to the event Xi.

#### Trajectories of gravity center transfer

Information on the evolution process of the spatial distribution of A-level scenic spots in Guangdong Province, and that on the overall displacement, were obtained after evaluating the trajectory and the law of the gravity center of the spatial distribution of A-level scenic spots in Guangdong Province each year. The theoretical formula for the gravity center is as follows:

$$X_{i}=\frac{\sum_{i=1}^{n}x_{i}}{n},\;Y_{i}=\frac{\sum_{i=1}^{n}y_{i}}{n}$$ (4)

In Eq (4), Xi and Yi represent the longitude and latitude coordinates of the distribution center of A-level scenic spot in Guangdong Province in a year. n represents the sum of the elements, which indicates the number of the A-level scenic spots in a year. xi and yi represent the longitude and latitude coordinates of the i-th scenic spot.

#### Standard deviation ellipse (SDE)

SDE reflects the spatial distribution characteristics of research elements and the spatial distribution changes and rules of the research elements by considering multiple angles [54]. Using the SDE method, the present study calculated the specific parameters of SDE of the spatial distribution of A-level scenic spots in each year, and further obtained information on the evolution process and law of spatial distribution of the A-level scenic spots in Guangdong Province.

#### Accessibility analysis

Accessibility can reflect the degree of difficulty between a region and other regions [55]. Therefore, the accessibility of the A-level scenic spots can be considered as the average travel time from one scenic spot to other scenic spots. The evaluation indices for accessibility are as follows:

$$R_{i}=\sum_{j=1}^{n}T_{ij}/n$$ (5)

where i and j represent the A-level scenic spots in Guangdong Province. Tij represents the shortest travel time from i to j through the transportation network. n represents the number of A-level scenic spots. Ri represents the average travel time between A-level scenic spots. The following can be implied: the smaller the value, the greater the accessibility to the scenic spot; hence, it is more convenient than other scenic spots.

## Results

### Evolution of spatial distribution characteristics of A-Level scenic spots in Guangdong Province

#### Analysis on the development and change of A-level scenic spots

**Fig 1:**
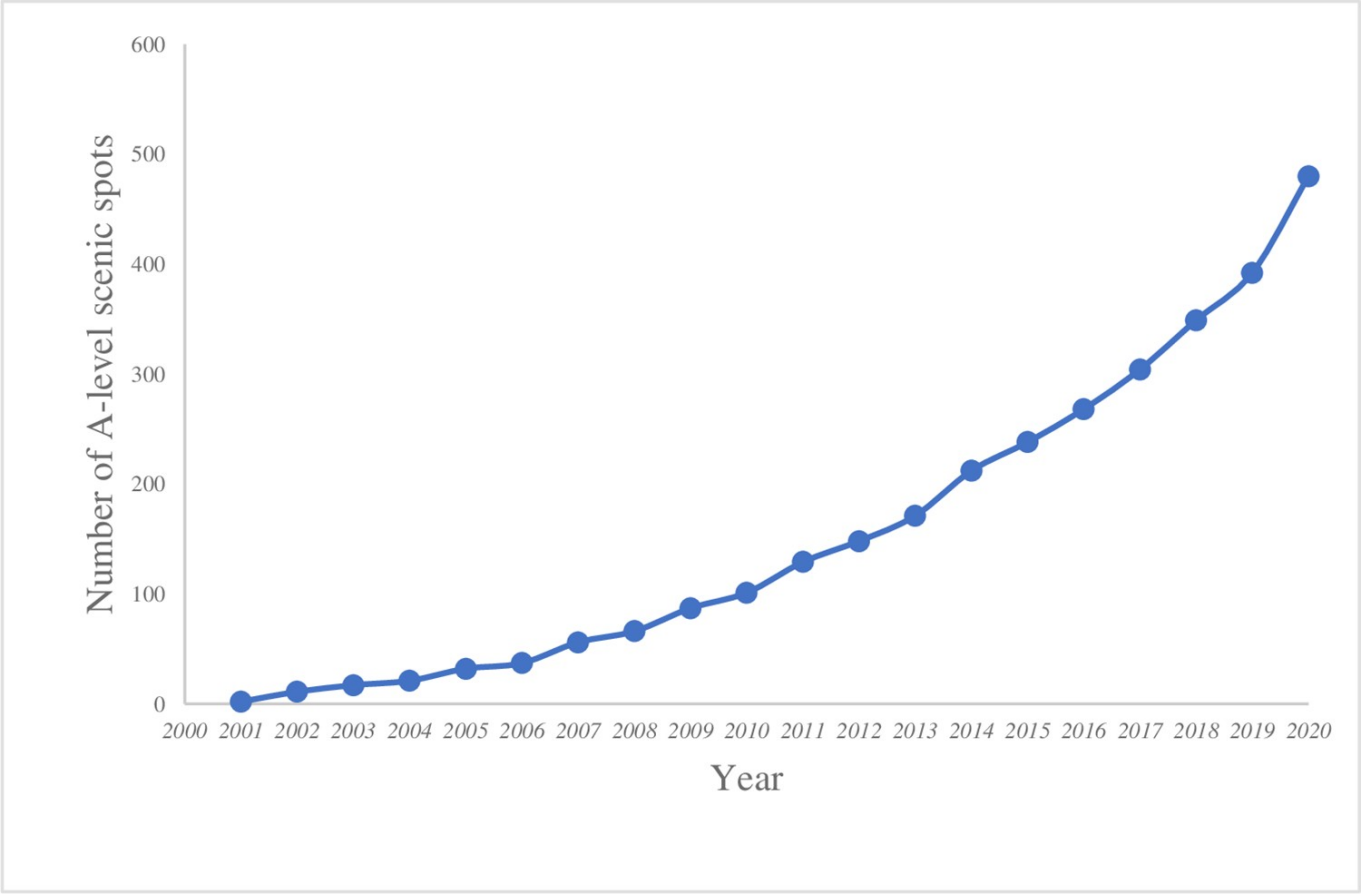
Transformation in the number of A-level scenic spots in Guangdong Province from 2001 to 2020.

Evolution of the number of A-level scenic spots in Guangdong Province (Fig 1) [56] since 2001 shows that the number of A-level scenic spots increased from 2 to 480, and the number continues to increase annually.

These data are available at: https://zenodo.org/record/5533146#.YVKLRcheW-W

The development process has been divided into three stages:

Stage 1: The initial development period (2001–2006). The National Tourism Administration initiated the implementation of an evaluation system for A-level scenic spots in 2001. The relevant evaluation system, policies, and standards at this time were in need of improvement. In the initial stage, the A-level scenic spots did not exhibit a brand effect.
Stage 2: The gentle development period (2007–2013). During this period, with the maturity of relevant policies, systems, and standards, the evaluation and the review of the A-level scenic spots had been standardized, and the brand effect of A-level scenic spots gradually gained prominence. The number of the A-level scenic spots in Guangdong Province increased steadily during this period.
Stage 3: The rapid development period (2014–2020). Owing to the economic transformation of Guangdong, the tertiary industry of tourism was developed. During this period, tourism emerged as an important industry for economic development in Guangdong. Compared with the first two stages, the increasing demand and preference of tourists considerably improved the quality and environment of scenic spots. The development of tourism also boosted the development of several upstream and downstream industries, resulting in the formation of a scale effect. Hence, the development of scenic spots has accelerated as it is an integral part of tourism industry. In 2014, the number of A-level scenic spots increased rapidly to 212, and the growth rate reached 41%. From this year onward, A-level scenic spots presented with rapid development.

#### Evolution of spatial distribution types

Using ArcGIS10.2, we analyzed the average nearest neighbor index of the A-level scenic spots in Guangdong Province annually. For each year, specific analysis results, including the actual nearest distance C_1_, the theoretical nearest distance C_2_, the nearest neighbor ratio C’, and the spatial distribution type (Table 1) were also obtained.

**Table 1:** Nearest neighbor analysis of spatial distribution of A-level scenic spots in Guangdong Province.

Year	Actual nearest distance/km	Theoretical nearest distance/km	Nearest neighbor ratio	Types of spatial distribution
2001	183.641001	162.522928	1.129939	Random
2002	50.387923	69.300009	0.727098	Clustered
2003	32.661146	55.744904	0.585904	Clustered
2004	33.33005	50.155664	0.664532	Clustered
2005	29.028182	40.630732	0.714439	Clustered
2006	25.232117	37.785813	0.667767	Clustered
2007	22.850979	30.713946	0.743994	Clustered
2008	18.570808	28.29161	0.656407	Clustered
2009	19.103797	24.641663	0.775264	Clustered
2010	18.625546	22.870147	0.814404	Clustered
2011	17.305449	20.236471	0.855161	Clustered
2012	17.251832	18.892907	0.913138	Clustered
2013	16.231525	17.576467	0.923481	Clustered
2014	13.332019	15.785622	0.844567	Clustered
2015	12.689223	14.898452	0.851714	Clustered
2016	11.811547	14.039843	0.841288	Clustered
2017	11.551263	13.204085	0.874825	Clustered
2018	10.486376	12.303167	0.852331	Clustered
2019	9.79939	11.608781	0.844136	Clustered
2020	8.782091	10.49081	0.837122	Clustered

It could be inferred from Table 1 that the main distribution type throughout the development process was cluster distribution. In 2001, the distribution was characterized as random; however, between 2002–2020, it was characterized as an agglomeration distribution and the degree of agglomeration changed minimally throughout this period.

The nearest neighbor ratio C’ in 2001 was greater than one, while from 2002–2020, it was less than one. This could be attributable to the fact that there were only two A-level scenic spots (Yuanming new gardens in Zhuhai City and Qingxin District in Qingyuan City) in Guangdong Province in 2001. Hence, C’ is greater than one because of the considerable distance between the scenic spots. Therefore, the distribution in 2002 was determined as a random distribution. Since 2002, the number of the A-level scenic spots in Guangdong Province developed significantly and the spatial distribution of the A-level scenic spots changed to an agglomeration type. From 2001 to 2003, the nearest neighbor ratio decreased from 1.13 to 0.58. Between 2004 and 2009, it showed a state of fluctuation. From 2010–2020, the value of C’ increased to more than 0.80. Since 2020, the value of C’ remained above 0.80, thereby showing relative stability.

#### Spatial distribution equilibrium

The number of scenic spots in each city is ranked from low to high; the proportion and the cumulative proportion of the number of the scenic spots in each city were calculated. Thereafter, the Gini coefficient of each year is calculated by substituting it into the Gini coefficient formula. The Gini coefficient is classified based on the type of the spatial distribution equilibrium represented by the Gini coefficient. The results are shown in Table 2.

**Table 2:** Grading results of Gini coefficient.

Year	Gini coefficient	Types of equilibrium degree
2001	0.904761905	Wide gap
2002	0.770562771	Wide gap
2003	0.722689076	Wide gap
2004	0.712018141	Wide gap
2005	0.68452381	Wide gap
2006	0.646074646	Wide gap
2007	0.476190476	Large gap
2008	0.493506494	Large gap
2009	0.401751505	Large gap
2010	0.372465818	Relatively reasonable
2011	0.400885936	Large gap
2012	0.394465894	Relatively reasonable
2013	0.414926204	Large gap
2014	0.387690925	Relatively reasonable
2015	0.390556222	Relatively reasonable
2016	0.358564321	Relatively reasonable
2017	0.31934622	Relatively reasonable
2018	0.316277801	Relatively reasonable
2019	0.316812439	Relatively reasonable
2020	0.316666667	Relatively reasonable

**Fig 2:**
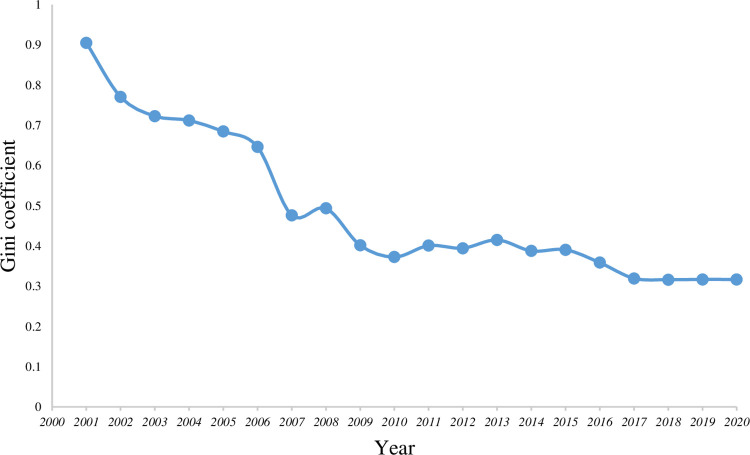
Transformation of Gini coefficient.

According to the Gini coefficient’s calculation results in Table 2, the following can be concluded:

1. The spatial distribution equilibrium of the A-level scenic spots in 21 cities in Guangdong Province tends to be "relatively reasonable." The Gini coefficient is not less than 0.3, and this shows that the spatial distribution is unbalanced every year. From the perspective of disequilibrium, the Gini coefficient was the greatest in 2001, indicating that the distribution of the Gini coefficient in this year was characterized by disparity. The Gini coefficient in 2018 is the smallest and closest to 0.3, indicating that the distribution of the year is the closest to the absolute average. Overall, there exist the following three types of equilibrium degrees from 2001 to 2020: "relatively reasonable," "large gap", and "wide gap." In the early development process of the A-level scenic spots "wide gap" was dominant but it has not been observed since 2007.
2. The evolution of the spatial distribution equilibrium can be divided into three stages and two turning points by analyzing the change in the Gini value of the Gini coefficient from 2001 to 2020 (Fig 2) Apart from the unique year of 2001, the three stages are 2002–2007, 2007–2015, and 2015–2020, and the two turning point years are 2007 and 2015.

In the first stage from 2002 to 2007, the Gini coefficient showed a yearwise decrease. The overall performance indicated that the value of the spatial distribution equilibrium increased and tended to be absolutely average. The evolution process of the spatial distribution equilibrium during this stage can be summarized as "high-low." In the second stage from 2007 to 2015, the Gini coefficient fluctuated, and the evolution process of the spatial distribution equilibrium value could be summarized as a "low-high-low-high" periodic change. In the third stage from 2015, the Gini coefficient decreased in a yearwise manner and reached its lowest value in 2018. The overall performance indicates that the value of the spatial distribution equilibrium continues to increase and tends to be absolutely average. The evolution process of the spatial distribution equilibrium can be summarized as "high-low." There are two turning points in 2007 and 2015. In 2007, the Gini coefficient of the spatial distribution of the A-level scenic spots in the 21 cities of Guangdong Province gradually increased, showing a fluctuating change. From 2015, the degree of the spatial distribution equilibrium gradually stabilized and inclined toward the establishment of an equilibrium.

### Evolution process of the spatial distribution of A-Level scenic spots in Guangdong Province

#### Density change process

The spatial distribution of the A-level scenic spot underwent noticeable changes in the nuclear density between 2001–2020, and demonstrated evident periodic characteristics as detailed below. The number of high-density areas increased continuously from 2001 to 2020. In 2001, Guangdong Province had only two A-level scenic spots, which were distributed in Zhuhai City and the Qingyuan fresh area. Therefore, the density of Guangdong Province in 2001 was depicted as two high-density cores. In 2002, the A-level scenic spots developed steadily, which was a remarkable improvement when compared to 2001 and was depicted as many high-density areas. These high-density areas were the primary areas of density change throughout the study period; thus, the distribution density of the A-level scenic spots has evolved into core areas, the majority of which are in the Pearl River Delta area and in four high-density distribution areas in the east, the west, and the north of Guangdong Province.

#### Center of gravity migration process

The center of gravity of the spatial distribution of A-level scenic spots and the geometric center of Guangdong Province were calculated by using the ArcGIS 10.2 software. Based on these results, the center of gravity of each year was superimposed with the geometric center of Guangdong Province. The azimuth angle, the east–west offset distance, the south-north offset distance, and the offset distance of each year’s center of gravity were calculated (Table 3).

**Table 3:** Center of gravity parameter for the spatial distribution of A-level scenic spots in Guangdong Province.

Year	Coordinates		Location	East-West migration distance (km)	South-North migration distance (km)	Offset distance (km)
2002	114.080°E	23.110°N	Buoluo, Huizhou	/	/	/
2003	113.160°E	22.628°N	Pengjiang, Jiangmen	102.408	57.860	117.623
2004	113.507°E	22.802°N	Nansha, Guangzhou	38.672	20.819	43.920
2005	113.298°E	22.849°N	Shunde, Foshan	23.312	5.657	23.988
2006	113.263°E	22.925°N	Shunde, Foshan	3.851	9.138	9.916
2007	113.190°E	22.942°N	Shunde, Foshan	8.093	2.019	8.341
2008	113.341°E	23.054°N	Haizhu, Guangzhou	16.783	13.558	21.575
2009	113.393°E	23.091°N	Haizhu, Guangzhou	5.769	4.441	7.280
2010	113.597°E	23.177°N	Zengcheng, Guangzhou	22.710	10.329	24.948
2011	113.623°E	23.248°N	Zengcheng, Guangzhou	2.886	8.587	9.060
2012	113.599°E	23.244°N	Zengcheng, Guangzhou	2.678	0.486	2.722
2013	113.658°E	23.278°N	Zengcheng, Guangzhou	6.603	4.103	7.774
2014	113.769°E	23.298°N	Zengcheng, Guangzhou	12.404	2.359	12.626
2015	113.813°E	23.300°N	Zengcheng, Guangzhou	4.819	0.199	4.823
2016	113.826°E	23.276°N	Zengcheng, Guangzhou	1.523	2.891	3.267
2017	113.815°E	23.241°N	Zengcheng, Guangzhou	1.285	4.228	4.419
2018	113.775°E	23.217°N	Zengcheng, Guangzhou	4.408	2.851	5.249
2019	113.743°E	23.205°N	Zengcheng, Guangzhou	3.560	1.463	3.849
2020	113.715°E	23.230°N	Zengcheng, Guangzhou	3.139	3.063	4.386

According to the migration track and the center of gravity metric (Table 3), the key observations are as follows:

1. The trajectory of the center of gravity movement initially moves west and then toward the east. From 2002 to 2007, the center of gravity was transferred from Boluo County, Huizhou City, through the Pengjiang District, Jiangmen City to Nansha District, and Guangzhou. In 2007, it migrated eastward progressively. In 2010, the center of gravity reached Zengcheng District, Guangzhou.
2. The distribution of the center of gravity is stable. From the perspective of the distribution of the center of gravity, the types of administrative areas where the center of gravity is located are limited. They are generally distributed in "five districts, four cities, and one county." The five districts are Zengcheng, Haizhu, Nansha, Shunde, and Pengjiang districts. The four cities include Guangzhou, Foshan, Huizhou, and Jiangmen, respectively. The county is Boluo County.
3. The range of the center of the gravity coordinate is not wide, which indicates that the level of a scenic spot is distributed intensively. Based on the analysis of the spatial distribution centers of the A-level scenic spots in each year, the center of the gravity coordinate from 2001 to 2020 was 113.159°E–114.080°E, 23.109°N–23.230°N. The range is not wide. Compared with the Guangdong geometric center (113.431°E, 23.347°N), the largest east–west migration occurred in 2003. The largest north–south migration also occurred in 2003. From the perspective of the migration distance, the distance between the center of gravity and the geometric center of Guangdong Province is minimal every year. In the east–west direction, the migration distance shows a trend of "decrease–increase–decrease." The maximum offset distance was 102.408 km in 2003, and the minimum offset distance was 1.285 km in 2005 and 2017. In the south-north direction, the migration distance shows a trend of "decrease–increase–decrease." The maximum offset distance was 191.941 km in 2003, and the minimum distance was 2.722 km in 2012.

### SDE analysis

An SDE analysis was conducted using the ArcGIS 10.2 software. The relevant parameters of SDE were calculated. The relevant parameters of SDE are listed in Table 4.

**Table 4:** Results of the standard deviation ellipses.

Year	X-axis standard deviation (km)	Y-axis standard deviation (km)	Ellipse area(km^2^)	Deflection angle θ(°)	Roundness
2002	80.598	235.142	59529.088	76.704	0.343
2003	80.566	359.499	90956.119	63.521	0.224
2004	82.801	367.504	95560.736	64.691	0.225
2005	84.766	352.101	93732.889	64.055	0.241
2006	93.142	330.484	96680.197	63.300	0.282
2007	93.223	295.687	86579.474	64.598	0.315
2008	96.201	292.924	88511.628	64.718	0.328
2009	102.255	276.360	88764.868	66.117	0.370
2010	110.053	280.695	97034.017	68.307	0.392
2011	120.408	269.701	102008.858	66.581	0.446
2012	123.096	260.069	100563.413	65.884	0.473
2013	124.119	256.928	100174.673	66.596	0.483
2014	124.066	260.148	101385.943	67.049	0.477
2015	120.495	263.225	99632.378	67.210	0.458
2016	119.015	262.657	98195.869	66.858	0.453
2017	118.127	262.407	97370.623	66.754	0.450
2018	120.381	261.672	98951.036	66.232	0.460
2019	119.868	254.475	95819.600	65.783	0.471
2020	119.325	254.403	95358.755	67.027	0.469

According to the results of SDE depicted in Table 4, the following conclusions can be deduced:

1. Though the SDE changes every year from 2002 to 2020, minimal change has been observed to the SDE shape, direction, orientation, and area of the ellipse. Simultaneously, it is noted that since 2002, the orientation of the ellipse has shown a trend of distribution along the "northeast-southwest" direction. This indicates that the spatial distribution orientation of the A-level scenic spots in Guangdong Province is similar to that of Guangdong Province.
2. The evolution process of the area of SDE is periodically "increase–decrease–increase–decrease." The spatial distribution characteristics are "dispersion–concentration–dispersion–concentration." Based on the change in the ellipse area, the minimum value of SDE area was inferred as 59529.088 km^2^ in 2002. The ellipse area continued to increase from 2007 to 2011, reaching a maximum value of 102008.858 km^2^. At this juncture, the distribution characteristics of the A-level scenic spots in Guangdong Province were observed to be the most dispersed.
3. The major axis of the SDE first increased and then decreased. This change in the major axis of the SDE indicates the change degree of the spatial distribution of A-level scenic spots in Guangdong Province along the main axis direction (east–west). With respect to the change of the long axis, the distance of the long axis was the smallest in 2002 (235.142 km). The greatest distance was reported in 2004 (367.504 km). The overall change in the long axis can be summarized as a process of "increase–decrease." From 2002 to 2004, since the A-level scenic spots were in the initial stage of the multi-regional distribution and development and in the stage of east and west expansion, the long axis of the ellipse showed a growing trend. Since 2005, the number of scenic spots in the Pearl River Delta region has increased significantly as compared to other regions in Guangdong. Therefore, the SDE gradually converged along the long axis of the northeast-southwest direction and the long axis of the ellipse showed gradual decrease again.
4. Overall, the short axis of the SDE shows an increasing trend. The change in the short axis (south-north) of the SDE indicates the degree of change of the spatial distribution of A-level scenic spots in Guangdong Province. The results indicated that the short axis distance was the smallest in 2003 (80.566 km) and was the greatest in 2013 (124.119 km). From 2002 to 2020, the SDE gradually expanded along the south-north direction.

### Analysis of accessibility results

According to the accessibility analysis, the accessibility values between all A-level scenic spots (480 in total), 2A scenic spots, 3A scenic spots, 4A scenic spots, 5A scenic spots, and data on the tourist source areas in Guangdong Province were obtained. They were interpolated using the inverse distance weight method, and the relevant data have been displayed in Table 5.

**Table 5:** Comparison of accessibility for 480 A-level scenic spots in Guangdong Province.

Node of the A-level scenic spot	Accessibility index		Node of the A-level scenic spot	Accessibility index	
	Time	Sort		Time	Sort
Chuanlord Tourism & Leisure EXPO Park	1.561	1	Yutang Fu Luogang Town	5.253	471
Chimelong Tourist Resort	1.592	2	PingShan Terrace	5.259	472
Guangzhou baiyun mountain scenic area	1.592	3	Maode gonggucheng Resort	5.267	473
Tourism Area of Sun Yat-sen Hometown	1.828	4	Maoming Xijiang Hot Spring Resort	5.272	474
Xiqiao Mountain Scenic Spot	1.864	5	Xiyan tea village attractions	5.283	475
Shennong Thatched Cottage Traditional Chinese Medicine Museum	1.934	6	Tianchi scenic spot of Fenghuang Mountain	5.514	476
OCT Travel and Resort Area	1.937	7	Maoming Fangji Island	5.866	477
GuangZhou Zoo	1.942	8	Maoming tianmashan ecotourism area	5.970	478
South China botanical garden	1.950	9	Gaozhou Xianren cave scenic area	6.031	479
Grandview Plaza	1.956	10	Leizhou Tianchengtai Resort	6.301	480

The accessibility of the 480 A-level scenic spots in Guangdong Province and county-level administrative regions in Guangdong Province ranges from 1.56 to 6.30 h. The average accessibility time is 3.14 h. Among them, 255 A-level scenic spots are within three hours, accounting for 53.1% of all A-level scenic spots. For all 2A scenic spots, the accessibility time value ranges from 2.27 to 3.92 h. The average accessibility time value is 3.16 h. There are seven 2A scenic spots for which the accessibility time value is less than three hours, accounting for 50.0% of all 2A scenic spots. For all 3A scenic spots, the accessibility time value ranges from 2.02 to 6.30 h. The average accessibility time value is 3.19 h. Among them, 138 3A scenic spots have an accessibility time value within three hours, accounting for 52.3% of all 3A scenic spots. For all 4A scenic spots, the accessibility time value ranges from 1.93 to 5.87 h. The average accessibility time value is 3.12 h. Among them, 99 4A scenic spots have an accessibility time value within three hours, accounting for 52.7% of all 4A scenic spots. For all 5A scenic spots, the accessibility time value ranges from 1.56 to 4.31 h. The average accessibility time value is 2.56 h. Among them, 10 5A scenic spots showed an accessibility time value of less than three hours, accounting for 71.4% of all 5A scenic spots.

Based on the statistical data, it was found that all top 10 A-level scenic spots with good accessibility were located in the cities of Guangzhou, Foshan, and Shenzhen in the Pearl River Delta region. This is because several influencing factors considered when calculating accessibility present with an increase in this region. In the Pearl River Delta region, the social and economic levels and the population are the highest within Guangdong Province. The 10 A-level scenic spots with the lowest accessibility value are located in the western and eastern Guangdong. In these areas, the social and economic levels and the population are low, which directly impacts the level of accessibility to A-level scenic spots in this area. These results show that the traffic network is only one of the factors affecting regional accessibility. The accessibility of the region is jointly affected by many factors. Based on the average accessibility value, the accessibility cost of the high-level scenic spots is smaller than that of low-level scenic spots. Based on the perspective of a low value ratio of the accessibility cost, the high-level scenic spot is also preferable than the low-level scenic spot, which shows that the connection between the grade of a scenic spot and the tourist source area in Guangdong Province is relatively close. Additionally, the close relationship between the high-level scenic spot and the tourist source area is stronger than that between the low-level scenic spot and the tourist source. The overall pattern of accessibility in Guangdong Province is similar to the spatial structure of the A-level scenic spots in Guangdong Province. Therefore, there is a concentration in the central area of the Pearl River Delta.

Generally, the area with a low accessibility value in the A-level scenic spots in Guangdong Province exhibits a centralized distribution. The distribution of the core area is concentrated in the Pearl River Delta region and is outwardly distributed. This area demonstrates the presence of many 5A scenic spots that are distributed intensively. The high social and economic levels and the large population of this area correlate with the good accessibility in this area. Simultaneously, the social economy, regional population, scenic area level, and other aspects exert a significant impact on the accessibility between the scenic spots and the tourist sources.

## Discussion and conclusions

In the present study, the spatial and temporal evolution characteristics of A-level scenic spots in Guangdong Province from 2001 to 2020 were analyzed. Furthermore, the spatial distribution characteristics, spatial distribution equilibrium, the evolution process, and the accessibility of the A-level scenic spots in Guangdong Province were analyzed using the methods of the metrology geography and GIS. The main conclusions are as follows:

1. A-Level scenic spots in Guangdong Province have undergone development in the following three stages from 2001 to 2020: the initial development period, gentle development period, and rapid development period. In the province, the spatial distribution has primarily shown the cluster type, and the degree of the agglomeration has remained high. The spatial distribution evolution and accessibility of A-level scenic spots in Guangdong Province are influenced by multiple factors, such as regional natural environment, population, and socio-economy, with high spatial and temporal heterogeneity.
2. The type of the spatial distribution equilibrium in 21 prefecture-level cities of Guangdong Province from 2001 to 2020 has gradually developed from a "wide gap" to a "relatively reasonable" type. Furthermore, 2018 showed a pattern that was the closest to the average distribution type. Specifically, the Gini coefficient declined from 2002 to 2007, fluctuated from 2007 to 2015, and decreased from 2015 to 2020. This indicated that with the steady growth in the number of the A-level scenic spots in Guangdong Province, the spatial distribution in the province gradually shifted from a high concentration in 2001 to a balanced development. The equilibrium degree of the spatial distribution gradually stabilized from 2015 onward. This is the result of different stages of economic development and tourism development policies.
3. The nuclear density map shows that the number and the range of high-density areas have continued to increase. A pattern of multiple high-density areas has formed, with the Pearl River Delta emerging as the main core. Five major scenic spots have evolved, namely the Pearl River Delta high-density area, the east high-density area, the west high-density area, and the north high-density area. This is related to the endowment of tourism resources, the level of economic development, the traffic conditions, the natural geographical environment, and population.
4. The center of the gravity migration track of the A-level scenic spots moved to the west and then to the east between 2002–2020. From 2005 to 2009, it gradually moved eastward, and in 2010, the center of gravity reached Zengcheng District, Guangzhou. The distribution of the center of gravity was stable throughout the study period. The orientation of the SDE shows a trend along the distribution direction of "northeast-southwest", indicating that the spatial distribution of A-level scenic spots in Guangdong Province is similar to the regional shape direction of the Province.
5. The regional accessibility of A-level scenic spots in Guangdong Province has improved during the study period. Moreover, the accessibility of the high-level scenic spots is better than that of low-level scenic spots. However, the area with the best accessibility demonstrates an evident aggregation phenomenon. The overall pattern is centered in the central region of the Pearl River Delta, and it exhibits an outer ring-shaped structure. This results from the fact that Guangzhou, the capital of Guangdong Province, is located in the Pearl River Delta region, with rapid urban development, a high level of urbanization, an obvious radiation-driven effect, and a long history of tourism development. The presence of a large population in this zone influences the scale of the scenic spots consumption market and travel preferences, and also influences the formation and distribution of A-level scenic spots in Guangdong Province.

According to the research conclusion, to optimize the spatial layout of scenic spots and to promote high-quality and sustainable development of tourism in Guangdong Province, we present the following suggestions: (1) At the provincial level, development of the whole area of tourism should be based on the provincial tourism development plan, guide the cities to use their strengths and complement their weaknesses, following the path of characteristic and differentiated tourism development. First of all, encourage cities to strengthen and upgrade the traditional industry while taking into account the new needs of tourists and the development of new industries to enrich the type of scenic spots, especially entertainment and shopping tourism scenic spots or projects. Second, improve the transportation network system, opening up transportation links between scenic spots and the last kilometer of transportation to scenic spots so as to facilitate the travel of tourists. Third, coordinate the construction of tourism infrastructure and service facilities to achieve regional sharing of facilities and seamless connection of tourism services, and realize the continuity of tourism services throughout the region. Fourth, expand marketing channels, improve the visibility of special brands, improve the level of information technology tourism and services, and build a first-class tourism service brand. (2) From the perspective of cities in Guangdong Province, cities should make full use of the agglomeration advantage brought by the tourism economic growth pole, which is a high-level tourism scenic spot or advantageous scenic spot. Based on the tourist demand brought by the growth pole, cities should use big data and multimedia to increase the number of creative tourism projects, improve tourism elements, and enrich the types of tourism scenic spots, thus building mutually supportive and complementary tourism development axes and eventually forming tourist destinations loved by tourists. (3) The agglomeration and distribution of A-level scenic spots in Guangdong Province are evident, and the balance is poor. The Pearl River Delta region should continue to play a leading role in promoting the rapid development of tourist spots in Eastern, Western, and Northern Guangdong provinces, and should gradually help establish a balance of the layout of A-level scenic spots in Guangdong Province. (4) The A-level scenic spots should be clarified with their own functional needs, with avoidance of homogeneity and disorderly competition, and facilitation of resource agglomeration and combination, to help realize the potential of tourism and to introduce tourism products and quality services to meet the market demand. For the areas with insufficient tourism resources, it is necessary to fully explore and innovate distinctive tourism resources and to implement differentiated competitive strategies. (5) There are some areas with poor accessibility to A-level scenic spots in eastern, western, and Northern Guangdong provinces; hence, investment in transportation facilities and the optimization of the traffic network structure should be prioritized in these areas [57].

The innovative aspect of this study is that it is an overall study of both the spatial distribution evolution and accessibility of A-level scenic spots in Guangdong Province from an intra-regional perspective, which is an enrichment and expansion of the study of the spatial distribution and accessibility of scenic spots and has certain academic significance. This study considers Guangdong Province in terms of the spatial distribution and evolution of A-level scenic spots from 2001 to 2020. We used metrology geography and GIS; therefore, other mathematical methods and theoretical analysis is relatively lacking. The qualitative analysis content is limited, and there is a lack of in-depth discussion of the reasons for the spatial distribution evolution of the A-level scenic spots. Furthermore, only the annual spatial distribution of A-level scenic spots was studied. Future development prospects and the spatial distribution of A-level scenic spots have not been predicted, and further research is warranted. This study calculated the accessibility of A-level scenic spots in Guangdong Province, without considering the accessibility of tourists arriving from outside Guangdong Province and also ignored the impact of air traffic. The methods of scientific exploration of the comprehensive accessibility of tourists to A-level scenic spots within and outside the province merits further in-depth study. Subsequent research should further deepen and expand the following aspects: (1) calculation of travel time in this study takes into account the ideal (average) situation of road traffic on weekdays, and does not take into account special situations such as traffic congestion; in the future, big data on urban traffic (such as cab track data) and POI data of A-level scenic spots can be introduced to combine big data methods with traditional geography methods; (2) spatial distribution differences can be used to clarify the advantageous tourism resources of cities in Guangdong Province, discover the growth poles of scenic spots, find the tourism development positioning, and propose corresponding tourism marketing strategies.

## Supporting information

S1 Fig Provincial map of China—Guangdong Province 13.5 million. (JPG)

S1 File List of A-level scenic spots in Guangdong Province. (XLSX)
