# Supplementary figures and images for: Spatial distribution evolution and accessibility of A-level scenic spots in Guangdong Province from the perspective of quantitative geography
Source: PLoS One. 2021 Nov 15;16(11):e0257400. doi: 10.1371/journal.pone.0257400 (PMC8592449; doi:10.1371/journal.pone.0257400)

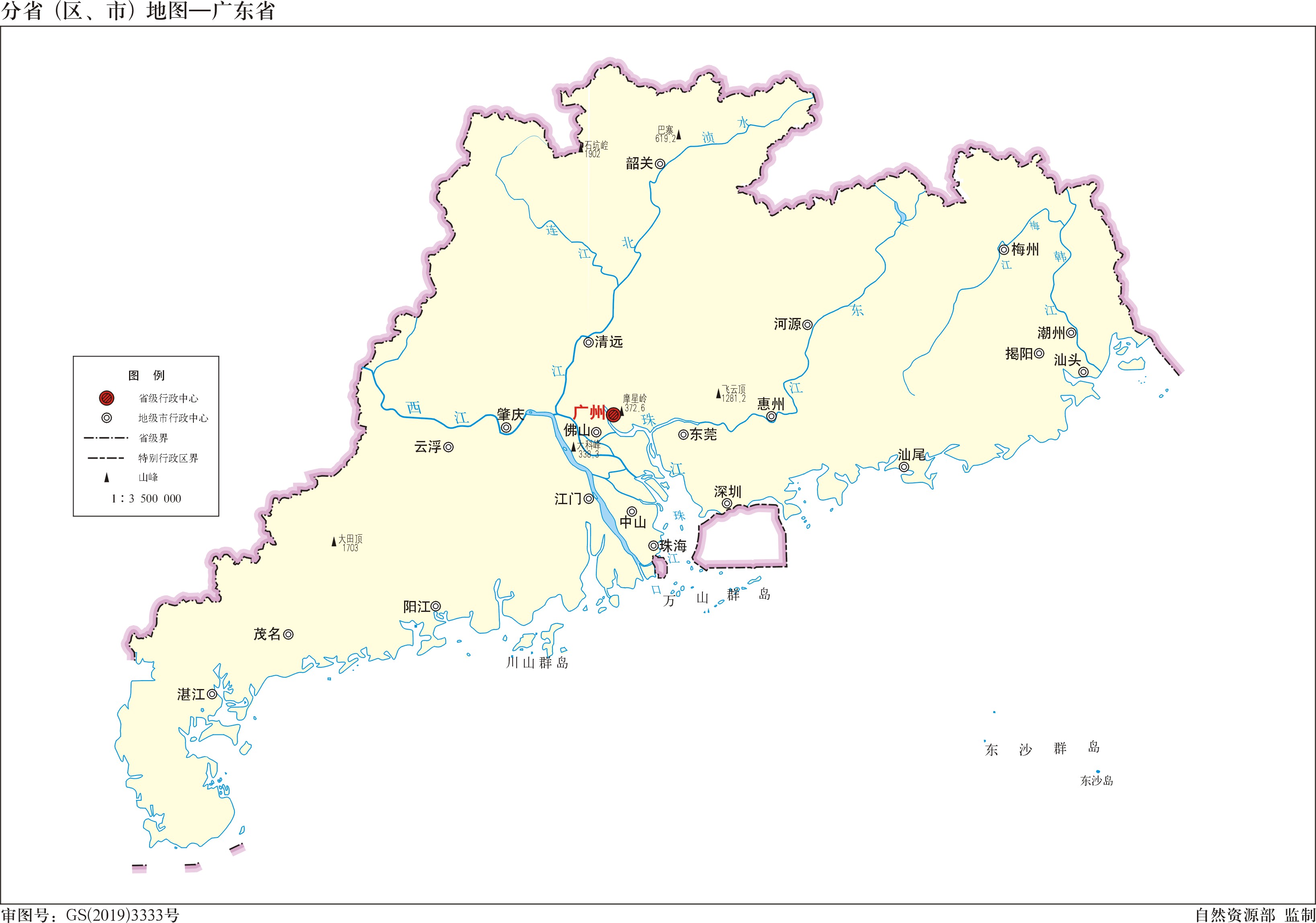

Supplement: S1 Fig — (JPG) [file pone.0257400.s001.jpg]
